# Assessment of intestinal luminal stenosis and prediction of endoscopy passage in Crohn’s disease patients using MRI

**DOI:** 10.1186/s13244-024-01628-5

**Published:** 2024-02-16

**Authors:** Wenjuan Wu, Yan Jin, Dongyang Zhu, Junqing Wang, Yue Cheng, Lei Zhang

**Affiliations:** 1 Department of Radiology, Wuxi Second People’s Hospital, Jiangnan University Medical Center, Wuxi, 214002 China; 2 Department of Gastroenterology, Wuxi Second People’s Hospital, Jiangnan University Medical Center, Wuxi, China

**Keywords:** Crohn disease, Magnetic resonance enterography, Endoscopy, Stenosis, Endoscopic passage

## Abstract

**Background:**

Crohn’s disease (CD) is an inflammatory disease of the gastrointestinal tract. The disease behavior changes over time, and endoscopy is crucial in evaluating and monitoring the course of CD. To reduce the economic burden of patients and alleviate the discomfort associated with ineffective examination, it is necessary to fully understand the location, extent, and severity of intestinal stenosis in patients with CD before endoscopy. This study aimed to utilize imaging features of magnetic resonance enterography (MRE) to evaluate intestinal stenosis in patients with CD and to predict whether endoscopy could be passed.

**Methods:**

MRE data of patients with CD were collected, while age, gender, disease duration, and laboratory test parameters were also gathered. Two radiologists analyzed the images and assessed whether endoscopy could be passed based on the imaging performance. Imaging features of MRE were analyzed in groups based on endoscopy results.

**Results:**

The readers evaluated the imaging performance for 86 patients to determine if endoscopy could be passed and performed a consistency test (compared between two readers *k* = 0.812, *p* = 0.000). In the univariate analysis, statistical differences were observed in the degree of T1WI enhancement, thickness of the intestine wall at the stenosis, and diameter of the upstream intestine between the two groups of whether endoscopy was passed. In multivariate logistic regression, the diameter of the upstream intestine was identified to be an independent factor in predicting whether endoscopy was passed or not (OR = 3.260, *p* = 0.046).

**Conclusions:**

The utilization of MRE signs for assessing the passage of an endoscope through the narrow segment revealed that the diameter of the upstream intestine emerged as an independent predictor of endoscopic passage. Before performing an endoscopy, MRE can aid in evaluating the passage of the endoscope.

**Critical relevance statement:**

This retrospective study explored the imaging features of MRE to evaluate intestinal stenosis in patients with Crohn’s disease and determined that the diameter of the upstream intestine of the stenotic segment was an independent predictor in assessing endoscopic passage.

**Key points:**

• Endoscopy is crucial in evaluating and monitoring the course of Crohn’s disease.

• The diameter of the upstream intestine of the stenotic segment was an independent predictor in assessing endoscopic passage.

• MRE can aid in evaluating the passage of the endoscope in stenotic segments of Crohn’s disease.

**Graphical Abstract:**

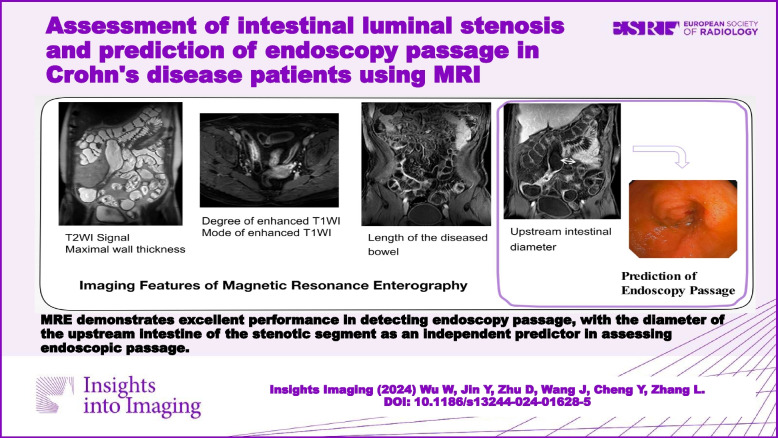

## Introduction

Crohn’s disease (CD), an inflammatory disease of the gastrointestinal tract, is characterized by chronic and relapsing inflammation of the mucous membrane and transmural lining of the intestine [1]. CD can involve any part of the gastrointestinal tract and is classified into three subtypes based on age, location of disease, and disease behavior (B1 non-stenosis non-penetrating, B2 stenosis, and B3 penetrating) [2]. The disease behavior changes over time, and severe inflammation can lead to fistulas or strictures [3]. However, intestinal stenosis has received limited attention, as it does not correlate well with the severity of the lesion and therapeutic outcome. According to the current European Crohn’s and Colitis Organisation (ECCO) guidelines, the diagnosis and monitoring of Crohn’s disease necessitate a confluence of clinical, imaging, and laboratory findings [4].

Endoscopy is crucial in evaluating and monitoring the course of CD [5, 6]. Endoscopy is typically conducted at the time of initial diagnosis, early on (usually 8–12 weeks after treatment initiation), and in the long term (usually 1 year). Furthermore, endoscopy may be warranted in the event of a significant alteration in the treatment regimen or to rule out active inflammation that may impact treatment choices [7]. However, if the intestinal cavity is narrow and endoscopy is difficult to pass, the examination cannot be performed. In order to alleviate the financial strain on patients and minimize unnecessary discomfort, it is imperative to comprehensively understand the location, extent, and degree of stenosis prior to conducting the endoscopy.

Cross-sectional enterography methods serve as a complement to ileocolonoscopy and can visualize intramural or proximal small bowel inflammation in around 50% of Crohn’s disease patients with normal endoscopic findings [8]. Based on the consensus guidelines [8], computed tomography enterography (CTE) and magnetic resonance enterography (MRE) are endorsed as the predominant imaging modalities for staging and surveillance of Crohn’s disease. MRE is a valuable imaging tool that accurately characterizes the manifestations of intraluminal and extraluminal diseases. MRE is preferred over CTE due to its lack of ionizing radiation [9]. The European Guidelines for the diagnosis and treatment of Crohn’s disease and colitis advocate for the utilization of MRE for the evaluation of intestinal strictures and patency in inflammatory bowel disease [10]. Despite the high prevalence of strictures in Crohn’s disease, there is a paucity of research on the rates of progression and the factors contributing to progression, which are essential for informing clinical decisions in pharmacologic therapy and surgery. Current imaging scoring systems consist of MARIAs, which assesses intestinal thickening, edema, fat creeping, and ulcers, and MEGS, which assesses intestinal wall thickening, diseased intestinal length, pattern and degree of TWI enhancement, and complications. These systems solely evaluate the level of inflammatory activity and do not consider the degree of intestinal stricture [11, 12]. Therefore, the purpose of this study was to utilize MRE features in evaluating intestinal stenosis in patients with CD and predicting whether endoscopy would pass.

## Materials and methods

### Patient selection and data collection

A retrospective analysis was conducted on patients diagnosed with CD at Wuxi Second People’s Hospital from March 2021 to May 2023. The diagnosis of CD was based on the Lennard–Jones criteria [13]. The study was approved by the Human Research Committee of our hospital, and informed consent was obtained from each patient. The inclusion criteria were as follows: (1) patients with a confirmed diagnosis of CD, (2) at least 18 years old, (3) undergoing MRE with lesions located in the jejunum and ileum, (4) comprehensive clinical and laboratory examination data available, (5) endoscopy performed within 1 week before and after MRE examination, and (6) no history of intestinal resection before MRE examination. The exclusion criteria were as follows: (1) pregnant women, (2) clinical remission period exceeding 3 years, (3) poor quality MRE images, and (4) presence of other intestinal tumors.

Data collection on each patient, including age, gender, disease duration, and laboratory test indicators, was performed using the Picture Archiving and Communication System (PACS), endoscopy examination database, and medical record system.

### MRE inspection methods and parameters

Before the scan, the patient fasted for 8 h and underwent intestinal cleansing with oral laxatives. Consumption of water was permitted. One hour before the scan, patients ingested 2.5% isotonic mannitol solution (Stone medicine Silver Lake Pharmaceutical Co., Ltd.) totaling 1600–2000 mL, with 400–500 mL given orally every 15 min for 4 doses. Five to 10 min after the final oral dose, scopolamine (654–2, Anhui Changjiang Pharmaceutical Co., Ltd.) 10 mg was administered via intramuscular injection, and the scan commenced 5–10 min later. MRE examination was performed using a Philips 3.0 T or Siemens 3.0-T scanner. The contrast agent used was gadopentetate acid dimeglumine salt injection (Magnevist, Schering Corporation), with a dose of 0.2 mL/kg and injection rate of 3 mL/s. The patient was positioned prone, the scan sequences included coronal T2-weighted imaging (T2WI), axial T2-weighted imaging (T2WI) with fat suppression, axial T1-weighted imaging (T1WI), diffusion-weighted imaging (DWI) (*b* = 0/800), and coronal dynamic contrast-enhanced sequences. The scan parameters are presented in Table 1.

**Table 1:** MRE image acquisition parameters

**Scan sequence**	**Plane**	**Philips 3.0 T (*n* = 74)**			**Siemens 3.0 T** (***n* = 12**)		
		**Slice width (mm)**	**FOV**	**TR/TE**	**Slice width (mm)**	**FOV**	**TR/TE**
T2WI	Coronal	5	420 × 467	2000/80	5	450 × 450	1200/122
T2WI	Axial	3	302 × 380	1800/90	3	380 × 380	1500/90
T1WI	Coronal	5	420 × 467	10/2.3	5	450 × 450	4.2/1.34
DWI	Axial	3	302 × 380	12,000/60.50	1.5	380 × 380	5600/55
DCE	Coronal	0	420 × 467	4.96/7	1.5	450 × 450	4.2/1.34

### MRE image analysis

MRE image analysis was conducted independently by two radiologists (reader 1 with 3 years of diagnostic experience in MRE and reader 2 with 15 years of diagnostic experience in MRE, certified by the unit board). The physicians were blinded to any clinical data or endoscopy results. They assessed whether the endoscopy would pass based on MRE image performance.

The senior radiologist also evaluated the radiographic imaging features of the CD from each MRE scan. T2WI signal of the small intestinal wall in the lesion was categorized as normal, mild increase, moderate increase, and severe increase (defined as intestinal luminal fluid signal). The degree of enhanced T1WI was classified as no enhancement, mild enhancement, moderate enhancement, and severe enhancement (defined as vascular enhancement). The mode of enhanced T1WI was categorized as homogeneous enhancement, mucosal enhancement, and stratified enhancement. Additional MRE imaging features included the length of the diseased intestine, intestinal wall thickness, and upstream intestinal dilatation diameter before stenosis.

### Grouping according to endoscopy results

For SES-CD, the four endoscopic factors chosen were ulcers, the proportion of surface area affected by ulcers, the percentage of surfaces with any other lesions, and stenosis. Each factor was scored 0 to 3 in each segment: ulcers were evaluated based on their size (0.1–0.5 cm, 0.5–2 cm, or > 2 cm in diameter), the proportion of ulcer surface with different extents of involvement (< 10%, 10–30%, or > 30%), the percentage of affected areas based on the degree (< 50%, 50–75%, or > 75%), and stenosis was classified as single or multiple and whether the endoscopy could be passed through the narrowed lumen [14]. Based on intestinal patency under endoscopy, the patients with CD were divided into three groups: group A without intestinal stenosis, group B with intestinal stenosis and passable endoscopy results, and group C with intestinal stenosis and non-passable endoscopy results. The combination of groups A and B constituted the endoscopy passable group.

### Statistical analysis

All statistical calculations were performed using the SPSS software (version 25; IBM Corp., Armonk, NY). Continuous variables were presented as mean ± standard deviation, while categorical variables were expressed as frequencies and percentages. Cohen’s kappa coefficient (intra-group correlation coefficient) was utilized to assess the agreement between the readers and endoscopy results for all measured parameters. The consistency between the two with *k* ≤ 0.2 is considered slight, with 0.4 < *k* ≤ 0.6 is considered moderate, with 0.6 < *k* ≤ 0.8 is considered substantial, and with *k* > 0.8 is considered perfect [15]. Patient characteristics between the groups were compared using the *t* test, the Mann–Whitney *U* test, or the Kruskal–Wallis rank sum test. After conducting univariate comparative analyses between the groups, the Youden index [16] was analyzed using receiver operating characteristic (ROC) curves to determine the cutoff values of continuous variables with a *p* value less than 0.1. Subsequently, the continuous variables were converted into ordered categorical variables. Multivariate logistic regression analysis was performed to identify independent factors affecting endoscopic passage, and odds ratio (OR) and 95% confidence interval (CI) were calculated. A *p*-value less than 0.05 was considered statistically significant.

## Results

### Characteristics of the study groups

**Fig. 1:**
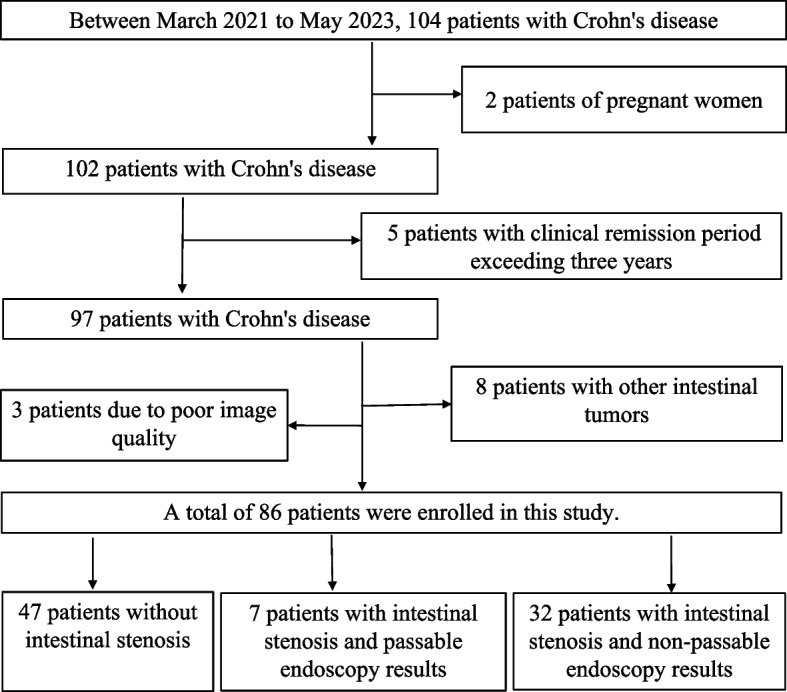
Flow diagram of the inclusion of patients in the study

The present study included 86 patients with a mean age of 34.01 years (± 11.38), and 65 of whom were male (Fig. 1). The mean BMI was 20.09 kg/m^2^ (± 2.67). Demographic data, baseline characteristics, and clinical outcomes for the study population are presented in Table 2. No statistical significance was observed between the groups about age, gender, BMI, disease duration, lesion location, and laboratory indices. Conversely, SES-CD scores were statistically significant among the three A, B, and C groups (*p* = 0.038), and there was also statistical significance between the two groups (between groups A + B and C) (*p* = 0.015), but there was no statistical significance between the two groups of B and C.

**Table 2:** Clinical parameters of patients

	**Group A** (***n* = 47**)	**Group B** (***n* = 7**)	**Group C** (***n* = 32**)	**Comparison of A, B, and C groups**		**Comparison between group A + B and group C**		**Comparison between group B and group C**	
				***F*/*X***^**2**^**/*H***	***p* value**	***t*/*X***^**2**^**/*H***	***p* value**	***t*/*X***^**2**^**/*H***	***p* value**
Age (years) ± SD	34.19 ± 11.73	30.43 ± 10.16	34.53 ± 11.31	0.380	0.685	0.324	0.747	0.884	0.383
Sex (*n*)				1.301	0.585	1.289	0.304	0.816	0.346
Male	37	6	22						
Female	10	1	10						
BMI (kg/m^2^) ± SD	21.09 ± 2.43	19.75 ± 2.72	19.39 ± 2.94	0.759	0.483	0.859	0.401	0.882	0.822
Disease duration (M) (M, IQR)	36 (14, 83)	36 (12, 120)	30 (13, 110)	0.028	0.986	0.238	0.812	0.163	0.871
Albumin (g/L) ± SD	41.04 ± 5.31	42.33 ± 2.93	42.90 ± 3.75	0.244	0.786	0.530	0.603	0.229	0.828
CRP (mg/L) ± SD	18.18 ± 12.46	13.43 ± 9.53	15.23 ± 10.79	0.101	0.905	0.152	0.881	0.236	0.823
SES-CD score (M, IQR)	4 (0, 9)	5 (1, 12)	9 (3, 11)	6.526	**0.038**	2.434	**0.015**	0.628	0.530

*SES-CD* Simplified Endoscopic Score for CD, *BMI* Body mass index, *CRP* C-reactive protein, *SD* Standard deviation, *IQR* Inter-quartile range

### MRE prediction for intestinal stenosis and passage of endoscopy

Readers evaluated whether endoscopy could be passed according to the image performance, and the consistency test was performed by comparing endoscopy results. The results of the test indicated *k* = 0.690 (*p* = 0.000) for reader 1 and *k* = 0.783 (*p* = 0.000) for reader 2. The result of the comparison between the two readers was *k* = 0.812 (*p* = 0.000).

**Fig. 2:**
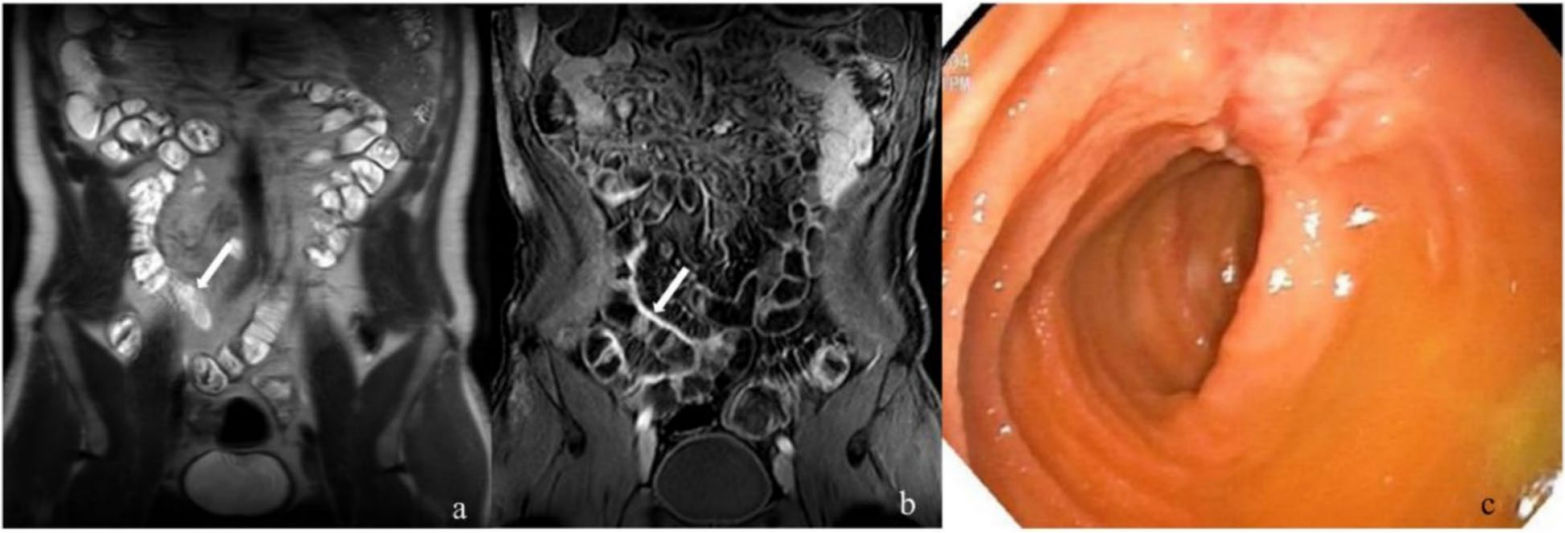
A 29-year-old man with Crohn’s disease. **a** Coronal T2-weighted imaging before endoscopy: *arrow* points the lesion in the terminal ileum with no significant thickening of the intestinal wall and mild hyperintensity. **b** Coronal enhanced T1-weighted imaging with moderate and homogenous enhancement (*white arrow*). **c** The endoscopy could pass

**Fig. 3:**
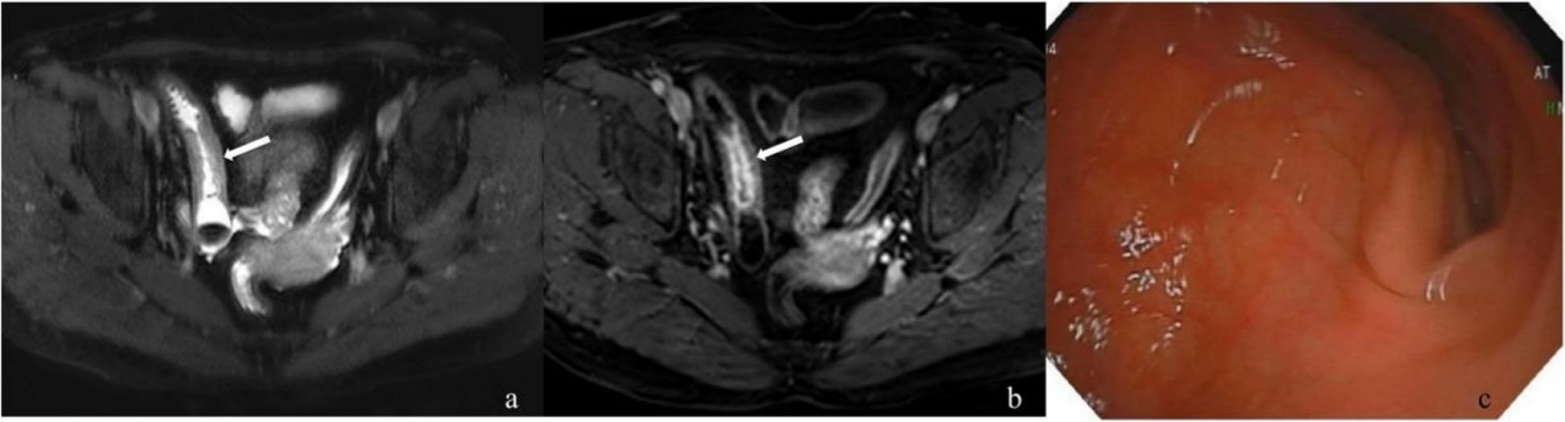
A 66-year-old woman with Crohn’s disease. **a** Axial T2-weighted imaging before endoscopy: *arrow* points the stenosis in the distal ileum with marked thickening of the intestinal wall and moderately high intensity. **b** Axial enhanced T1-weighted imaging with severe intestinal wall enhancement and mucosal enhancement (*white arrow*). **c** The endoscopy showed a narrow ileum, but the endoscopy could pass

**Fig. 4:**
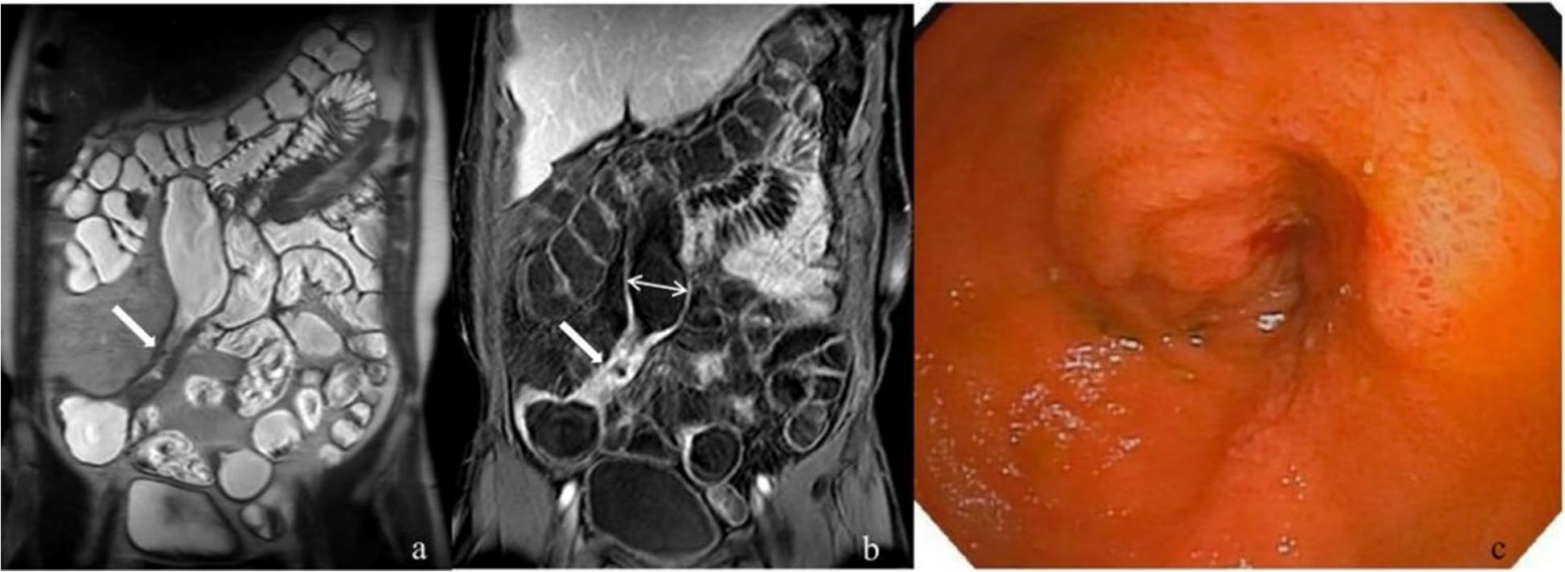
A 37-year-old man with Crohn’s disease. **a** Coronal T2-weighted imaging before endoscopy: marked thickening of the intestinal wall, narrowing of the intestinal cavity, and normal signals (*white arrow*). **b** Coronal enhanced T1-weighted imaging with dilation of the upper intestinal cavity (*double-ended white arrow*) and moderate and homogenous enhancement of the intestinal wall (*white arrow*). **c** The endoscopy showed a narrow ileum, but the endoscopy could not pass

No statistical differences were observed in T2WI signal, degree of enhanced T1WI, mode of enhanced T1WI, and length of diseased intestine among the A, B, and C groups. However, statistical significance in intestinal wall thickness and upstream intestinal diameter was observed between group A + B and group C (Table 3, Figs. 2, 3, and 4).

**Table 3:** Comparison of MRE signs between the groups

	Group A (*n* = 47)	Group B (*n* = 7)	Group C (*n* = 32)	Comparison of A, B, and C groups		Comparison between group A + B and group C		Comparison between group B and group C	
				***F*/*Z***	***p* value**	***t*/*Z***	***p* value**	***t*/*Z***	***p* value**
T2WI signal (*n*)				5.519	0.219	1.401	0.508	1.099	0.661
Normal	16	1	9						
Mild increase	26	3	15						
Moderate increase	5	3	8						
Degree of enhanced T1WI (*n*)				7.166	0.104	6.419	**0.036**	0.945	0.704
Mild enhancement	27	3	9						
Moderate enhancement	17	4	19						
Severe enhancement	3	0	4						
Mode of enhanced T1WI (*n*)				4.245	0.329	5.355	0.059	2.255	0.413
Homogeneous enhancement	27	3	9						
Mucosal enhancement	17	4	19						
Stratified enhancement	3	0	4						
Length of the diseased bowel (cm) ± SD	8.87 ± 7.18	12.60 ± 9.64	14.78 ± 19.75	1.866	0.161	1.812	0.074	0.282	0.780
Maximal wall thickness (mm) ± SD	6.22 ± 2.17	6.86 ± 2.87	7.70 ± 2.73	3.486	**0.035**	2.570	**0.012**	0.735	0.467
Upstream intestinal diameter (mm) ± SD	18.64 ± 7.29	19.29 ± 8.12	23.38 ± 7.34	4.384	**0.015**	2.969	**0.004**	1.446	0.157

Using the endoscopy result as a test criterion, ROC curve analysis was used to derive the cutoff values for the cumulative length of the diseased intestine, intestinal wall thickness at the maximum stenosis, and diameter of the upstream intestine, which were determined to be 14.3 cm, 5.15 mm, and 20.5 mm, respectively.

### Independent predictors with logistic regression

Binary logistic regression analysis was performed to assess whether the degree of enhanced T1WI, mode of enhanced T1WI, the length of the diseased bowel, the thickness of the bowel wall, and the diameter of the upstream intestine were independent predictors of endoscopy failure. The Box-Tidwell method was used to test for linearity, revealing a linear relationship between the logit-transformed values of all continuous independent variables and the dependent variable. Among the five independent variables assessed, the diameter of the upstream intestine was found to be an independent predictor of endoscopy failure (*p* < 0.05). The risk of endoscopy failure was 3.260 times higher when the diameter of the upstream intestine was > 20.5 mm compared to when it was < 20.5 mm (Table 4).

**Table 4:** Logistic regression analysis of independent factors

	**OR value**	**95% CI**	***p* value**
Degree of enhanced T1WI			
Mild enhancement	1		
Moderate enhancement	2.506	0.831–7.557	0.103
Severe enhancement	5.113	0.440–59.398	0.192
Mode of enhanced T1WI			
Homogeneous enhancement	1		
Mucosal enhancement	3.737	0.854–16.346	0.080
Stratified enhancement	0.949	0.057–15.834	0.971
Length of the diseased bowel	0.638	0.175–2.327	0.496
Maximal wall thickness	3.510	0.798–15.449	0.097
Upstream intestinal diameter	3.260	1.019–10.430	**0.046**

## Discussion

This retrospective investigation sought to assess the efficacy of MRE in detecting intestinal stenosis and predicting endoscopy passage of CD patients. The findings revealed statistical differences in T1WI enhancement, intestinal wall thickness, and upstream intestinal diameter between the groups based on endoscopy passage or failure. Remarkably, the upstream intestinal diameter emerged as an independent predictor of endoscopic passage failure.

Stenosis is a significant clinical concern, with approximately 10% of Crohn’s patients without stenosis at baseline developing symptomatic stenosis at 5-year follow-up, and more than half of CD patients progressing to significant intestinal obstruction during the clinical course [17–19]. The etiology of the continued progression may be attributed to incomplete inhibition of mucosal inflammation and mechanisms of intestinal damage unrelated to inflammation. CD patients require multiple endoscopies during long-term follow-up, but severe stenosis can limit or prevent the effectiveness of this procedure [5, 20–22]. In patients with stenosis, endoscopy can only reach the proximal end of the stenosis, making it impossible to judge the length of the lesion, the distal end of the stenosis, ulceration, and whether it has penetrated to form a fistula. Previous research [23] has demonstrated the challenge of accurately diagnosing small intestinal stenosis solely relying on patients’ clinical symptoms or laboratory indicators. Therefore, non-invasive imaging techniques such as MRE are necessary to evaluate stenosis before endoscopy. In this study, two radiologists assessed stenosis and predicted endoscopic passage with high consistency. Thus, it can be inferred that irrespective of diagnostic experience, radiologists can subjectively evaluate intestinal patency through MRE.

Subsequently, we investigated which MRE features validate endoscopic passage and will lead to better predictions in the future. Prior research has documented the sensitivity and specificity of MRE in detecting stenosis, ranging from 75 to 100% and 91 to 96%, respectively [24–26]. In terms of stricture characteristics, major strictures, extended strictures, and prestenotic dilation were identified as prognostic factors for stricture detection by MR imaging [27]. Our study found significant differences in T1WI enhancement, maximal wall thickness, and upstream intestinal diameter between the groups where the endoscope could be passed and where it could not through univariate analysis. However, no significant variations in these parameters were observed between the group with stenosis found to be passable by the endoscope and the group with stenosis found to be impassable, indicating that endoscopy can still pass through some intestinal lumens despite the presence of stenosis. In such a scenario, if the signs of stenosis on the MRE are entirely relied upon to determine endoscopy passage, there may be instances where some patients could potentially miss the diagnostic benefits of having an endoscopy. Compared with previous studies [25–30], this study not only found strictures, but also predicted whether the endoscopy would pass. Further analysis is imperative to establish a threshold for endoscopic passage based on MRE indicators.

In our study, logistic regression analysis was utilized to predict endoscopy passage using MRE signs. Out of the five signs that were assessed, the upstream intestine diameter was found to be statistically significant with a threshold of 20.5 mm for identifying endoscopy passage. A previous study [29] has indicated that the imaging manifestations of inflammation or fibrosis were inadequate in evaluating the severity of stenosis, and the expansion of the upstream intestine may indicate the severity of stenosis to some extent. Bettenworth et al. [30] have proposed a threshold value of 25 mm for the diameter of the upstream intestine for stenosis assessment. Nevertheless, the lower threshold observed in our investigation could potentially be attributed to a higher proportion of patients with ileus undergoing CT scans as opposed to MRI scans, so ileus patients with significant intestinal dilatation were not included in this study.

Moreover, the results revealed a significant difference in endoscopy SES-CD scores between the endoscopy passable and impassable groups, validating the progressive nature of CD driven by chronic inflammation. According to a longitudinal study spanning 20 years [31], around 60% of individuals diagnosed with Crohn’s disease initially exhibited B1 inflammatory disease, but 42% of them experienced a progression to a more widespread and intricate form of the illness. The presence of inflammatory or fibrous stenosis alone is exceedingly uncommon, with these factors nearly always co-occurring. The activation of mesenchymal cells, accumulation of extracellular matrix, smooth muscle hyperplasia in the mucosal and lamina propria layers, and scarring contribute to the eventual development of stenosis. Therefore, it is imperative to consider inflammation, fibrosis, smooth muscle hyperplasia, and stenosis from a clinical perspective [32–34].

However, the present study has several limitations. Firstly, its retrospective nature and single-center design may give rise to potential selection bias, particularly in relation to the severity of the disease. More severe cases of CD may be associated with failure to pass endoscopy. Secondly, the radiologist’s prediction of endoscopy success relied solely on qualitative evaluation based on clinical experience, without incorporating quantitative parameters due to the limited availability of relevant previous studies. Lastly, the incorporation of data from various MR scanners from different companies may have impacted the enhancement results, although this serves to validate the generalizability of the study findings.

In summary, the utilization of MRE signs for assessing the passage of an endoscope through the narrow segment revealed that the diameter of the upstream intestine emerged as an independent predictor of endoscopic passage. This discovery indicates that before performing an endoscopy, MRE can aid in evaluating the passage of the endoscope. In the future, MRE may offer new parameters for a deeper exploration of different phenotypes of CD.

## Data Availability

The datasets generated and/or analyzed during the current study are not publicly available due to patient privacy regulations but are available from the corresponding author upon reasonable request.

## Abbreviations

CD: Crohn’s disease
CI: Confidence interval
CTE: Computed tomography enterography
DWI: Diffusion-weighted imaging
MRE: Magnetic resonance enterography
OR: Odds ratio
PACS: Picture Archiving and Communication System
ROC: Receiver operating characteristic
SES-CD Score: Simplified Endoscopic Score for CD
T1WI: T1-weighted imaging
T2WI: T2-weighted imaging
